# Plasmid-mediated quinolone resistance genes transfer among enteric bacteria isolated from human and animal sources

**DOI:** 10.3934/microbiol.2021013

**Published:** 2021-06-09

**Authors:** EHWARIEME Daniel Ayobola, WHILIKI Onoriadjeren Oscar, EJUKONEMU Francis Ejovwokoghene

**Affiliations:** 1 Department of Microbiology, Faculty of Science, Delta State University, Abraka, Nigeria; 2 Department of Science Laboratory Technology, Delta State Polytechnic, Otefe-Oghara, Nigeria

**Keywords:** enteric bacteria, quinolones, gene transfer, minimum inhibitory concentration

## Abstract

This research investigates the transferability of plasmid-mediated quinolone resistance (PMQR) genes among enteric bacteria isolates in human and animal samples, as well as its implication on resistance of recipient cells. A total of 1,964 strains of five different enteric bacteria species (*Escherichia coli, Salmonella sp., Shigella sp., Klebsiella sp*. and *Aeromonas sp*.) were screened for plasmid-mediated quinolone resistance (PMQR) genes from a population of quinolone resistant (Q-r) isolates. Screening for PMQR isolates was achieved by plasmid curing using sub-lethal concentration of Sodium Dodecyl Sulphate and PMQR genes (*qnrA*, *qnrB*, *qnrS, Aac(6′)-Ib-cr*and *Qep A*) were detected by polymerase chain reaction (PCR). Conjugation and transformation experiments were attempted to ascertain transfer of genes from the Q-r isolates to a susceptible, standard recipient, *E. coli* J53-2. The minimum inhibitory concentration (MIC) was determined before and after gene transfer, using E-test strips. Results indicate that percentage resistance to the quinolones (Qs): Nalidixic acid, Ciprofloxacin, Pefloxacin and Ofloxacin determined by agar plate diffusion technique stood at 52.6, 47.3, 50.5, 70.6 and 46.0% for *Escherichia coli, Salmonella* sp., *Shigella*sp., *Klebsiella sp*. and *Aeromonas sp*. respectively. Analysis of variance indicated the occurrence of significant differences (F, 46.77–613.30; 0.00) in the resistance to each tested Qs. Generally, Human isolates showed greater resistance than Animal isolates (57.4 vs 47.2%). Investigation with specific primers indicated 11, 15, 7, 1 and 0 for *qnrA, qnrB, qnrS, qepA* and *Aac(6′)-Ib-cr* genes respectively, out of 1018 Q-r and 29 PMQR isolates. Gene transfer experiments indicated the transfer of all genes except *qepA* either by conjugation or transformation. The MIC of tested Qs on recipient bacterium before gene transfer greatly increased from 0.0625 to 0.25 µg/mL, after transfer. This study demonstrates that PMQR genes amongst enteric bacteria in the Niger delta of Nigeria were transferable and transfer conferred a higher Q- resistance on recipient bacterium.

## 1. Introduction

Pathogenic enteric bacteria are the major cause of food and water borne gastrointestinal illnesses in both humans and animals and remain a major public health problem the world over. Quinolones are the choice antibiotics for the treatment of various infections caused by pathogenic enteric bacteria [1]. However, widespread use of these antibiotics in human and veterinary medicine has led to the emergence of antibiotics resistance. Resistance to quinolones can lead to treatment failure and it is of public health concern [2].

Bacteria acquire resistance to quinolones by either chromosomal mutation-which occurs relatively slowly, confers high–level resistance and transmitted vertically, or by acquisition of plasmids containing genes for quinolone resistance. Plasmid-mediated quinolone resistance (PMQR) confers low- level resistance, but can be transmitted horizontally among members of the same and different species making their spread much faster than that of chromosomal mutation [3]. Plasmid-mediated quinolone resistance genes have been found in variety of Enterobacteriaceae especially *Escherichia coli, Enterobacter sp., Klebsiella sp. and Salmonella sp* [4]. Resistance to nalidixic acid and ciprofloxacin was reported as extremely high in isolates from broilers in European countries [5].

Transfer of antibiotics resistance genes (ARGs) plays an important role in the development of multidrug resistance (MDR) in bacteria [6]. There are three mechanisms of horizontal gene transfer in bacteria: conjugation, transduction and transformation [7]. Different studies have demonstrated that resistance genes could be transferred from one bacterium to the other both *in vitro* and *in vivo*. Transfer of resistance genes between *Shigella sp*. and *E.coli* [8],[9], *in vitro* intra and inter–species gene transfer of ampicillin resistance genes among enteric bacteria of diarrhea origin [10], transfer of tetracycline resistance determinants among bacteroids and other enteric bacteria in the human colon [11] have all being reported.

PMQR provide a low-level of quinolone resistance not reaching the clinical breakpoints defined by the Clinical and Laboratory Standard Institute criteria [12]. However, PMQR is clinically important because it facilitate the selection of higher levels of resistance. The combination of plasmid-mediated resistance with chromosomally encoded resistance mechanisms of drug classes now result in strains that are resistant to all of the main classes of commonly used antimicrobial drugs.

Enteric pathogenic bacteria are of particular interest because of their resistance to multiple antibiotics and their ability to transfer plasmids to other species and bacteria genera. It is therefore important to conduct a study on the transfer of plasmid mediated quinolone resistance gene among enteric bacteria isolated from human and animal sources. The aim of this study is to demonstrate the *in vitro* transferability of quinolone resistance determinants among enteric bacteria isolated from human and animal sources in Delta State, Nigeria where Self-medication and abuse of antibiotics are common.

## 2. Methods

### 2.1. Isolation of plasmid-mediated quinolone resistant enteric bacteria

A total of 1,964 strains of five different enteric bacteria species (*Escherichia coli, Salmonella sp., Shigella sp., Klebsiella sp*. and *Aeromonas sp*.) isolated from a previous study composed of 720 human and animal samples and comprises 180 diarrheal stools of patients attending public hospitals, 180 diarrheal stools of patients attending private hospitals, 180 poultry litters and 180 fish pond water samples collected from various hospitals patients, poultry and fish farms in major cities in Delta State, Nigeria. The isolates were screened for PMQR genes from a population of quinolone resistant (Q-r) isolates. The number of strains isolated from each source is shown in Table 1 [13]. Screening for PMQR isolates was achieved by plasmid curing using sub-lethal concentration of Sodium Dodecyl Sulphate and PMQR genes (*qnrA*, *qnrB*, *qnrS, Aac(6′)-Ib-cr* and *Qep A*) were detected by PCR [13]. All bacteria strains were isolated between September 2017 and December 2018.

### 2.2. Transfer of Quinolone Resistance

Conjugation and transformation experiments were performed on all 29 PMQR isolates to examine the ability to horizontally transfer resistance to quinolones conferred by *qnrA, qnrB, qnrS, aac(6′)-Ib* or *qepA* genes. Conjugation experiments involving the PMQR-positive isolates detected in this study were performed by the liquid mating assay [14]. Rifampicin resistant *E. coli* J53-2, azide-resistant Az ^r^ was used as the recipient strain and Luria-Bertani (LB) agar plates containing rifampicin 100 µg/mL, sodium azide 100 µg/mL, ciprofloxacin 20 µg, or nalidixic acid 20 µg/mL (Sigma-Aldrich LP, USA) were used for selection as required.

Transformation experiments were equally performed for PMQR-positive isolates. Plasmid DNA was extracted from the donor strains and introduced into chemically competent *E. coli* J53-2Az^r^ (Invitrogen, USA).Transformants were selected on Luria-Bertani plates supplemented with sodium azide 100 µg, rifampicin 100 µg/mL and nalidixic acid 20 µg/mL for selection as required.

#### 2.2.1 Conjugation experiment

The donor and recipient cells, both previously grown to exponential phase, were mixed in a ratio 1:9 (0.5 mL of the donor was added to 4.5 mL of the recipient in LB broth) and the incubation was continued at 37 °C, with very slow shaking (50 rpm). After 60 minutes, samples were withdrawn and diluted in 0.9% saline solution to 10^−1^, 10^−2^, 10^−5^, and 10^−6^.

One hundred (100) µL aliquots from dilutions 10^−1^ and 10^−2^ were spread on LB agar plates selecting for transconjugants and 100 µL aliquots from dilutions 10^−5^ and 10^−6^ were spread on LB plates selecting for donors and on LB plates selecting for recipients. Plates were incubated at 37 °C over night and colonies were counted and thereafter, conjugation frequencies were calculated by dividing the number of transconjugants by the number of donors. Transconjugants were inoculated on LB agar plates containing ciprofloxacin/nalidixic acid and sodium azide/rifampicin, to screen for transconjugants that co-acquired FQ and sodium azide resistance.

#### 2.2.2 Transformation experiment

For each transformation, one 50 µL vial of One Shot® *E. coli* J53-2 cells (Invitrogen, USA) was thawed on ice. Then, 2 µL of isolated plasmid was pipetted directly into the vial of competent cells and mixed by tapping gently. The vial was incubated on ice for 30 minutes. The vial was then incubated for 30 seconds at 42 °C in Eppendorf thermomixer device and 250 µL of pre-warmed S.O.C medium was added to the vial and subsequently incubated at 37 °C for 1 hour with shaking at 225 rpm. 50 µL from the transformation vial was spread on labeled LB agar plate supplied with the selecting antibiotics. The plate was incubated at 37 °C overnight and the number of colonies growing on the overnight plate was counted. Transformants were inoculated on LB-plates containing ciprofloxacin/nalidixic acid and sodium azide/rifampicin to screen for transformants that co-acquired ciprofloxacin and sodium azide resistance.

### 2.3. Sequencing the PMQR-positives

Sequencing was performed in this study by using the BigDye v3.1 sequencing chemistry (Applied Biosystems, USA). To confirm the identification of PMQR genes detected by PCR screening, same primers used in the multiplex and simple PCR screening were used for sequencing both strands of the detected genes [15].

Procedure:

The positive PCR products were purified and used as template for sequencing. Sequencing master mix:

### 2.4. Reagent amount

 Big-Dye v3.1 (Applied Biosystems, USA): 2 µL

 Sequencing buffer (Applied Biosystems, USA): 3 µL

 Primer 3.2 pmol/µL: 1 µL

 ddH_2_O: 12 µL

For each reaction, 2 µL template was added to 18 µL master mix and the PCR thermocycler was programmed. Nucleotide sequencing was performed at the Sequencing core facility of the International Institute for Tropical Agriculture (IITA), Ibadan.

Searching for nucleotide sequence homology was performed using the Basic Local Alignment Search Tool (BLAST) available at the National Center for Biotechnology Information website (http://www.ncbi.nlm.nih.gov/BLAST). Using the BLAST, the ultimate confirmation of gene sequences was made. First, an ExPasy convert to the amino acid sequence was done. All base and amino acid sequences were then used for a search in the database for homology of sequences. The BLAST-n and BLAST-p were used for nucleotide and amino-acid alignments, respectively.

### 2.5. MIC determination

The Minimum Inhibitory Concentration (MIC) of tested Qs on recipient bacterium before, and after gene transfer was equally determined using e-test strips.

## 3. Results

There is a significance difference in the occurrence of quinolone resistance among the isolates from the different sources. Except for *Klebsiella sp*., Q-resistance was generally higher amongst isolates from Public Hospitals (67.2%) than Private Hospitals (45.0%). Also, with the exception of *Salmnonella sp*., Q-resistance was generally higher amongst isolates from Poultry Litter (54.2%) than from Fish-pond Water (40.9%). As a composite, human isolates had a higher Q-resistance (57.4%) than Animal isolates (47.2%) and only 1018 of the 1964 isolated enteric bacteria strains were quinolone resistant as shown in Table 1.

**Table 1.** Number of Q-resistant strains (%) from each site according to species [13].

Species	Public Hospitals	Private Hospitals	Poultry Droppings	Fish Pond Water	TOTAL
*E.coli*	123/168	67/177	101/165	61/159	352/669
	(73.2)	(37.9)	(61.2)	(38.4)	(52.6)
*Salmonella*sp.	87/126	43/87	65/177	36/98	231/488
	(69.0)	(49.4)	(36.7)	(36.7)	(47.3)
*Shigella*sp.	77/131	38/76	29/46	18/68	162/321
	(58.8)	(50.0)	(63.0)	(26.5)	(50.5)
*Klebsiella*sp.	28/44	19/27	48/61	47/69	142/201
	(63.6)	(70.4)	(78.7)	(68.1)	(70.6)
*Aeromonas*sp.	21/31	11/29	31/56	68/169	131/285
	(67.7)	(37.9)	(55.4)	(40.2)	(46.0)

Total	336/500	178/396	274/505	230/563	1018
	(67.2)	(44.9)	(54.2)	(40.9)	

**Table 2.** Occurrence of PMQR amongst Q-resistant isolates from human and animal sources [13].

Species	Public Hospitals	Private Hospitals	Poultry Droppings	Fish Pond Water	TOTAL
*E.coli*	1/123	1/67	5/101	2/61	9/352
	(0.8)	(1.5)	(5.0)	(3.3)	(2.6)
*Salmonella*sp.	0/87	0/43	4/65	3/36	7/231
	(0.0)	(0.0)	(6.2)	(8.3)	(3.0)
*Shigella*sp.	0/77	0/38	1/29	2/18	3/162
	(0.0)	(0.0)	(3.4)	(11.1)	(1.9)
*Klebsiella*sp.	1/28	1/19	4/48	1/47	7/142
	(3.6)	(5.3)	(8.3)	(2.1)	(4.9)
*Aeromonas*sp.	0/21	0/11	2/31	1/68	3/131
	(0.0)	(0.0)	(6.5)	(1.5)	(2.3)

Total	2/336	2/178	16/274	9/230	29
	(0.6)	(1.1)	(5.8)	(3.9)	

**Table 3.** Resistance profile of plasmid-mediated quinolone resistant (PMQR) before and after plasmid curing.

PMQR Isolates	Resistance Profile Before Plasmid Curing				Resistance Retained After Curing
	NalidixicAcid (NA)	Ciprofloxacin (CPX)	Pefloxacin (PEF)	Ofloxacin (OFL)	
*E.coli* A067	R	R	R	R	NIL
*E.coli* A023	R	R	R	R	NIL
*E.coli* A031	R	R	R	R	NIL
*E.coli* A078	R	R	R	R	NIL
*E.coli* A033	R	R	R	R	NIL
*E.coli* A041	R	R	R	R	OFL
*E.coli* A051	R	R	R	R	OFL
*E.coli* H022	R	R	R	R	OFL
*E.coli* H015	R	R	R	R	NIL
*Salmonella* A010	R	R	R	S	NIL
*Salmonella* A089	R	R	R	S	NIL
*Salmonella* A035	R	R	R	R	NIL
*Salmonella* A030	R	R	R	R	NIL
*Salmonella* A022	R	R	R	R	NIL
*Salmonella* A001	R	R	R	S	NIL
*Salmonella* A031	R	R	R	R	NIL
*Shigella* A015	R	R	R	R	OFL
*Shigella* A031	R	R	R	R	NIL
*Shigella* A001	R	R	R	R	NIL
*Klebsiella* A029	R	R	R	S	NA
*Klebsiella* A022	R	R	R	S	NIL
*Klebsiella* A012	R	R	R	S	NA
*Klebsiella* A023	R	R	R	R	NIL
*Klebsiella* A033	R	R	R	R	OFL
*Klebsiella* H012	R	R	R	S	NIL
*Klebsiella* H022	R	R	R	R	OFL
*Aeromonas* A029	R	R	R	S	NA
*Aeromonas* A022	R	R	R	S	NIL
*Aeromonas* A012	R	R	R	S	NA

Key: NIL-Non-existence, OFL-Ofloxacin, NA-Nalidixic acid, R-Resistant, S-Susceptible

The ability to successfully transfer PMQR genes into the standard *E.coli^Az-r^* J53-2 recipient by conjugation and transformation was investigated. This was to ascertain whether the PMQR gene(s) is/are borne on transferable plasmids. This is crucial to the spread of resistance among bacterial population. Among the 5 *E.coli* strains detected with at least one of the PMQR gene (Table 4), conjugational transfer of at least a gene, was possible in four (80%). However, transfer by transformation of the competent recipient, was possible in all five transformation experiments.

All 7 of the *Salmonella* strains with PMQR genes in this study, were subjected to inter-generic transfer of resistance genes. Four (57.1%) of the transfer was possible by conjugation (Table 4), while six (85.7%) PMQR gene transfers were possible by transformation. The only *qepA* gene detected in this study in *Salmonella* A030, was not successfully transferred either by conjugation or transformation.

Inter-generic transfer of PMQR genes was equally attempted for the 2 *Shigella sp* investigated in this study. Gene transfer by conjugation was not successful though the recipient was successfully transformed with at least a PMQR gene being transferred (Table 4).

The 3 *Klebsiella sp*. positive for at least a PMQR gene studied, were equally subjected to inter-generic gene transfer by conjugation and transformation. None of the conjugation experiments was successful, but all three transformation experiments positively transferred at least one of the PMQR genes (Table 4).

Also attempted, was the inter-generic transfer of PMQR genes from the 2 *Aeromonas sp*. harboring at least a gene (Table 4). It was observed here again, that transfer by conjugation was not successful although at least a PMQR gene was positively transferred by transformation. Transformation was possible in 18/19 of the resistance transfer experiments while conjugation was only possible in 8/19 cases (Table 4). Generally, the *qnr*B and *qnr*S genes were always jointly transferred either by conjugation or transformation.

**Table 4.** Transfer of Plasmid bearing PMQR gene using *E. coli* J53-2 as recipient.

PMQR Donors	*PMQR Gene In Isolate	Gene(s) Transferred by	
		Conjugation	Transformation
*E.coli* A 067	QnrA	-	QnrA
*E.coli* A 023	QnrA, QnrS	QnrA	QnrA
*E.coli* A 031	QnrB, QnrS	QnrB, QnrS	QnrB, QnrS
*E.coli* A 078	QnrA, QnrB, QnrS	QnrB, QnrS	QnrB, QnrS
*E.coli* H 015	Qnr A, Qnr B	Qnr B	Qnr B
*Salmonella* A 010	QnrS	QnrS	QnrS
*Salmonella* A 089	QnrA, QnrB	QnrB	QnrB
*Salmonella* A 035	QnrB, QnrS	-	QnrB, QnrS
*Salmonella* A 030	QepA	-	-
*Salmonella* A 022	QnrB	QnrB	QnrB
*Salmonella* A 001	QnrA, QnrB	QnrB	QnrB
*Salmonella* A 031	Qnr B, Qnr S	-	QnrB, QnrS
*Shigella* A 031	Qnr A, Qnr B	-	Qnr B
*Shigella* A 001	Qnr B, Qnr S	-	Qnr B, Qnr S
*Klebsiella* A 022	Qnr A, Qnr B	-	Qnr B
*Klebsiella* A 023	Qnr A, Qnr B	-	Qnr A, Qnr B
*Klebsiella* H 012	Qnr B	-	Qnr B
*Aeromonas* A 041	Qnr A, Qnr B	-	Qnr B
*Aeromonas* A 033	Qnr A, Qnr B	-	Qnr A, Qnr B

Total 19	19	8	18

The Minimum Inhibitory Concentration (MIC) of Nalidixic acid(NA), Ciprofloxacin (CPX), Pefloxacin (PEF) and Ofloxacin (OFL) on the recipient bacteria (*E. coli* J53-2) lacking any PMQR gene/plasmid, was determined. The MICs were 0.25 µg/mL and 0.0625 µg/mL for NA and CPX respectively, and 0.0625 µg/mL and 0.031 µg/mL for PEF and OFL respectively. The respective PMQR gene was subsequently transferred to the recipient by transformation, and the MIC again determined. This was to ascertain the level of resistance conferred by the acquisition of PMQR plasmid(s).

**Table 5.** Minimum Inhibitory Concentration (MIC) of nalidixic acid on recipient bacteria, before and after PMQR gene transfer.

Isolates	PMQR Gene Transferred	MIC (µg/mL)		
		Donor	Recipient (Before Gene Transfer)	Recipient (After Gene Transfer)
*Aeromonas A-041*	Qnr B	64	0.25	4.0
*Aeromonas A-033*	Qnr A, Qnr B	128	0.25	16.0
*E.coli A-067*	Qnr A	32	0.25	2.0
*E.coli A-023*	Qnr A	128	0.25	16.0
*E.coli A-031*	Qnr B, Qnr S	128	0.25	16.0
*E.coli A-078*	Qnr B, Qnr S	128	0.25	16.0
*E.coli H-015*	Qnr B	64	0.25	4.0
*Klebsiella A-022*	Qnr B	64	0.25	4.0
*Klebsiella A-023*	Qnr A, Qnr B	64	0.25	16.0
*Klebsiella H-012*	Qnr B	32	0.25	4.0
*Salmonella A-010*	Qnr S	64	0.25	4.0
*Salmonella A-089*	Qnr B	64	0.25	8.0
*Salmonella A-035*	Qnr B, Qnr S	128	0.25	16.0
*Salmonella A-022*	Qnr B	32	0.25	4.0
*Salmonella A-001*	Qnr B	64	0.25	4.0
*Salmonella A-031*	Qnr B, Qnr S	128	0.25	16.0
*Shigella A-031*	Qnr B	64	0.25	8.0
*Shigella A-001*	Qnr B, Qnr S	128	0.25	16.0

**Table 6.** Minimum Inhibitory Concentration (MIC) of ciprofloxacin on recipient bacteria, before and after PMQR gene transfer.

Isolates	PMQR Gene Transferred	MIC (µg/mL)		
		Donor	Recipient (Before Gene Transfer )	Recipient (After Gene Transfer)
*Aeromonas A-041*	Qnr B	4.00	0.0625	2.00
*Aeromonas A-033*	Qnr A, Qnr B	8.00	0.0625	4.00
*E.coli A-067*	Qnr A	1.00	0.0625	1.00
*E.coli A-023*	Qnr A	2.00	0.0625	2.00
*E.coli A-031*	Qnr B, Qnr S	8.00	0.0625	4.00
*E.coli A-078*	Qnr B, Qnr S	8.00	0.0625	8.00
*E.coli H-015*	Qnr B	2.00	0.0625	1.00
*Klebsiella A-022*	Qnr B	2.00	0.0625	1.00
*Klebsiella A-023*	Qnr A, Qnr B	8.00	0.0625	8.00
*Klebsiella H-012*	Qnr B	2.00	0.0625	1.00
*Salmonella A-010*	Qnr S	1.00	0.0625	1.00
*Salmonella A-089*	Qnr B	2.00	0.0625	2.00
*Salmonella A-035*	Qnr B, Qnr S	8.00	0.0625	4.00
*Salmonella A-022*	Qnr B	1.00	0.0625	1.00
*Salmonella A-001*	Qnr B	2.00	0.0625	1.00
*Salmonella A-031*	Qnr B, Qnr S	8.00	0.0625	4.00
*Shigella A-031*	Qnr B	2.00	0.0625	2.00
*Shigella A-001*	Qnr B, Qnr S	8.00	0.0625	8.00

**Table 7.** Minimum Inhibitory Concentration (MIC) of pefloxacin on recipient bacteria, before and after PMQR gene transfer.

Isolates	PMQR Gene Transferred	MIC (µg/mL)		
		Donor	Recipient (Before Gene Transfer)	Recipient (After Gene Transfer)
*Aeromonas A-041*	Qnr B	4.00	0.0625	4.00
*Aeromonas A-033*	Qnr A, Qnr B	8.00	0.0625	2.00
*E.coli A-067*	Qnr A	1.00	0.0625	1.00
*E.coli A-023*	Qnr A	2.00	0.0625	4.00
*E.coli A-031*	Qnr B, Qnr S	8.00	0.0625	4.00
*E.coli A-078*	Qnr B, Qnr S	8.00	0.0625	8.00
*E.coli H-015*	Qnr B	2.00	0.0625	2.00
*Klebsiella A-022*	Qnr B	2.00	0.0625	2.00
*Klebsiella A-023*	Qnr A, Qnr B	8.00	0.0625	4.00
*Klebsiella H-012*	Qnr B	2.00	0.0625	2.00
*Salmonella A-010*	Qnr S	1.00	0.0625	2.00
*Salmonella A-089*	Qnr B	2.00	0.0625	4.00
*Salmonella A-035*	Qnr B, Qnr S	8.00	0.0625	4.00
*Salmonella A-022*	Qnr B	1.00	0.0625	2.00
*Salmonella A-001*	Qnr B	2.00	0.0625	4.00
*Salmonella A-031*	Qnr B, Qnr S	8.00	0.0625	4.00
*Shigella A-031*	Qnr B	2.00	0.0625	2.00
*Shigella A-001*	Qnr B, Qnr S	8.00	0.0625	4.00

**Table 8.** Minimum Inhibitory Concentration (MIC) of ofloxacin on recipient bacteria, before and after PMQR gene transfer.

Isolates	PMQR Gene Transferred	MIC (µg/mL)		
		Donor	Recipient (Before Gene Transfer)	Recipient (After Gene Transfer)
*Aeromonas A-041*	Qnr B	2.00	0.031	1.00
*Aeromonas A-033*	Qnr A, Qnr B	4.00	0.031	2.00
*E.coli A-067*	Qnr A	1.00	0.031	1.00
*E.coli A-023*	Qnr A	2.00	0.031	1.00
*E.coli A-031*	Qnr B, Qnr S	4.00	0.031	2.00
*E.coli A-078*	Qnr B, Qnr S	4.00	0.031	2.00
*E.coli H-015*	Qnr B	2.00	0.031	2.00
*Klebsiella A-022*	Qnr B	2.00	0.031	2.00
*Klebsiella A-023*	Qnr A, Qnr B	2.00	0.031	1.00
*Klebsiella H-012*	Qnr B	1.00	0.031	1.00
*Salmonella A-010*	Qnr S	1.00	0.031	1.00
*Salmonella A-089*	Qnr B	2.00	0.031	1.00
*Salmonella A-035*	Qnr B, Qnr S	4.00	0.031	2.00
*Salmonella A-022*	Qnr B	1.00	0.031	1.00
*Salmonella A-001*	Qnr B	2.00	0.031	1.00
*Salmonella A-031*	Qnr B, Qnr S	4.00	0.031	4.00
*Shigella A-031*	Qnr B	2.00	0.031	2.00
*Shigella A-001*	Qnr B, Qnr S	4.00	0.031	4.00

It was observed (Table 5) that transfer of PMQR plasmid resulted in an increase in MIC of NA from 3-(0.25–2.00 µg/mL), to 6-times (0.25–16.00 µg/mL) in the recipient bacterium. Also, PMQR gene transfer resulted in an increase in MIC of CPX and PEF from 4-(0.0625–1.00 µg/mL), to 7-fold (0.0625–8.00 µg/mL) in the recipient bacterium (Tables 6 and 7). Furthermore, transfer of PMQR plasmid to the recipient bacterium resulted in an increase in MIC of OFL from 5-(0.031–1.00 µg/mL), to 7-fold (0.031–4.00 µg/mL) as observed in Table 8.

**Figure 1.**
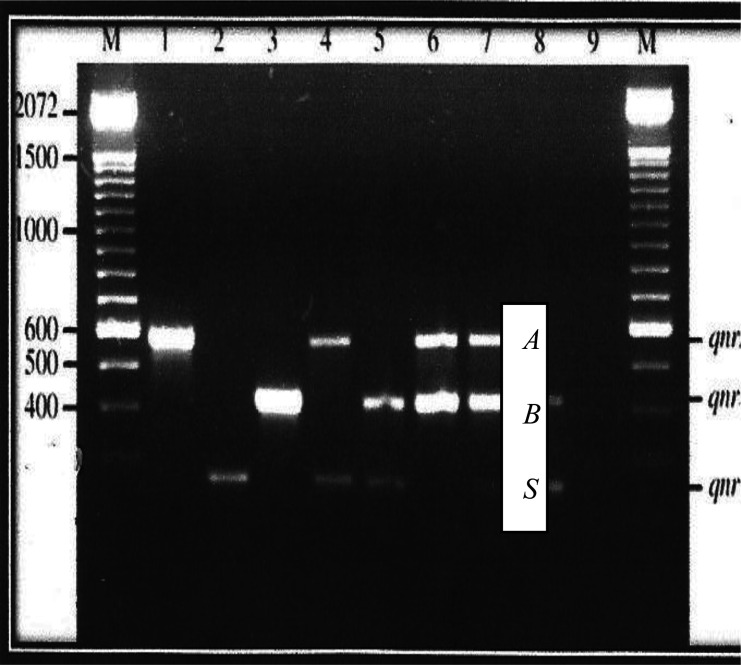
Plasmid bands and their approximate molecular weights of Plasmid-Mediated Resistant Isolates [13].

**Figure 2.**
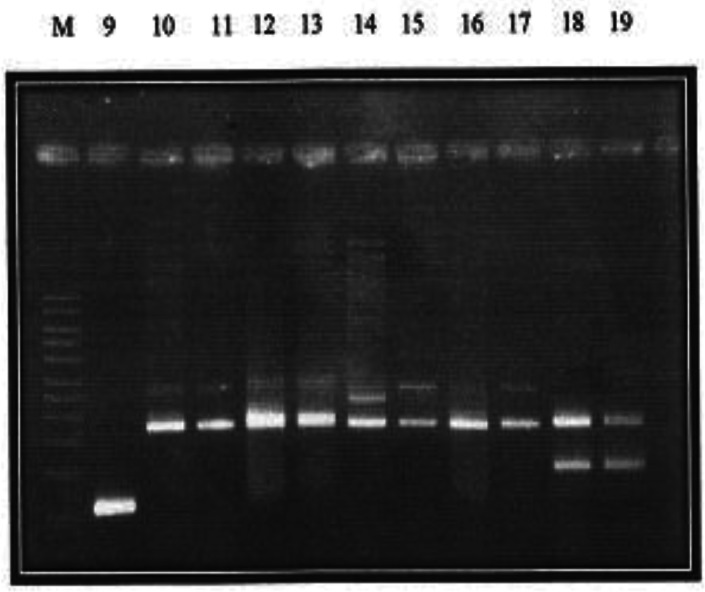
Plasmid bands and their approximate molecular weights of Plasmid-Mediated Resistant Isolates [13].

Presented in figures 1 and 2 are the plasmid bands of purified PCR products of all 19 isolates harboring PMQR genes. Plasmid sizes of bands were compared with the standard molecular weight ladder (M) at the extreme left of the plate.

The *qnr*A gene can be observed in lane 1 while lanes 3 and 16 display the *qnr*B gene. Along lane 2 is the *qnr*S gene, while the only *qep*A gene is located on lane 9. The corresponding approximate sizes of the *qep*A, *qnr*A, *qnr*B and *qnr*S genes are 199, 417, 469 and 516 bp, respectively (Figure 1). Lane 7 harbors three genes- *qnr*A, *qnr*B and *qnr*S while *qnr*A and *qn*rS gene bands are located on lane 4.The lanes 5, 8, 18 and 19 harbor both *qnr*B and *qnr*S genes. Furthermore, located along lanes 6, 10, 11, 12, 13, 14, 15 and 17 are PCR product bands for *qnrA* and *qnrB* genes.

Characterization of PMQR genes according to numbers showed that strains from which plasmids were extracted, on lanes 1, 2, 3 and 16 harbored just one (1) PMQR gene. Strains on lanes 4, 5, 6, 8, 9, 10, 11, 12, 13, 14, 15, 17, 18 and 19 harbored 2 PMQR genes. Only the strains on lane 7 harbored 3 PMQR plasmid bands.

In this study, a 100% alignment with BLAST-n was observed for *qnr*A gene, with *qnr*A database accession numbers KC 414000.1, JN 687470.1, JF 969163.1, JF 773308.1 and HQ 184955.1 belonging to isolates of *Enterobacter hormaechei, Providencia stuartii, Vibrio fluvialis, E. coli* and *Enterobacter cloaca* respectively. Furthermore, using the BLAST-p for amino acid alignment, a 99–100 % alignment was observed for qnrA gene in this study with those of *Klebsiella pneumoniea* isolates with accession numbers ABQ 01122.1, YP 002332855.1, ADO 60956.1, ADO 60962.1 and ADY 17943.1. Similar observations were recorded for the other PMQR determinants amplified in this study, by PCR.

## 4. Discussion

Results indicate the possibility of a high frequency of transfer of PMQR determinants, both by conjugation and transformation. Generally, transformation was possible in 18/19 of the resistance transfer experiments while conjugation was only possible in 8/19 cases. These results are quite significant to the dissemination of quinolone resistance in the environment. Also worthy of note is that, these transfers were not only intra-generic, but also inter-generic. However, inter-generic transfer by conjugation was unsuccessful in most cases.

Success in transferring the *qnr* genes by conjugation has been frequently reported [16],[17]. In addition, transfer of these *qnr* genes can also be performed by transformation [18]. On the other hand, both of *qnrS* and *qnrB* genes have also been detected on non-conjugative plasmids [19],[20]. In the study conducted by Jiang *et al*, southern hybridization indicated that *qnrA*, *aac(6′)-Ib-cr*, and ESBL-encoding genes were always located on the same plasmids [21]. However, the association between these genes on same plasmids was not confirmed in the present study.

On the other hand, the *qepA* gene were neither transconjugable nor transformable. The failure in transferring this gene suggests that it is located on a non-transferable plasmid. Nevertheless, a transferable plasmid has previously been demonstrated to carry the *qep* determinant; a plasmid which co-transfers the *rmt*B gene [22]. However, in the present study, it is difficult to draw conclusions regarding its non-transferability, because it was only in a single isolate that *qep* determinant was detected.

Interestingly, chromosomal location for *qnr* genes has been suggested by some studies [23],[19]. This could be the case for *Salmonella* A-030 in the present study, where repeated conjugation and transformation experiments yielded negative results. However, the observation was that *qnr*B and *qnr*S were always jointly transferred either by transformation or conjugation. This suggests that both genes are closely linked, or even occurring on the same plasmid. The acquisition of such plasmids bearing more than one Q-resistance marker could be detrimental epidemiologically. This may result in novel bacteria with multi-drug resistance capability which is not good for management of infectious diseases.

The high rate of PMQR gene acquisition by transformation, as observed in this study, is very significant. This is because, though transformation was induced and cells made competent in the present study, natural occurrences of transformation is quite possible [24]. In the natural world, DNA usually becomes available by death and lysis of other cells, but in the laboratory it is provided by the researcher, often as a genetically engineered fragment or plasmid. During uptake, DNA is transported across the cell membrane and the cell wall if one is present. Once the DNA is inside the cell it may be degraded to nucleotides, which are reused for DNA replication and other metabolic functions. Alternatively it may be recombined into the cell's genome by its DNA repair enzymes. If this recombination changes the cell's genotype the cell is said to have been transformed. Hence, for bacteria in diverse microbial pool, antibiotic resistance may so easily be disseminated among the microbial population [7].

It was observed that the transfer of PMQR plasmid resulted in an increase in MIC of Nalidixic acid from 0.25 up to 16.00 µg/mL in the recipient bacterium. Also, PMQR gene transfer resulted in an increase in MIC of Ciprofloxacin and Pefloxacin from 0.06 up to 8.00 µg/mL in the recipient bacterium. Furthermore, transfer of PMQR plasmid to the recipient bacterium resulted in an increase in MIC of Ofloxacin from 0.031 to 4.00 µg/mL). All observations pre-suppose that acquisition of the PMQR gene resulted in an increased resistance to the respective Q. The significant observation at this point is that, resistance status conferred by the acquisition of resistance plasmids did not at any instance, exceed the original resistance of the donor. This observation suggests that inasmuch as resistance is conferred by PMQR plasmid acquisition, the plasmid alone does not confer maximum resistance status. Other mechanisms of resistance, such as chromosomal mutation synergistically confer the maximum resistance possible. In other words, though plasmid-mediated resistance is important, it works hand-in-hand with chromosomally- mediated resistance, to pose an even more significant resistance status.

In general, the acquisition of a *qnr*-bearing plasmid will not render a wild-type organism quinolone insusceptible according to CLSI clinical breakpoints. The extent to which *qnr*A protects isolates of *Enterobacteriaceae* against quinolones has usually been examined by measuring the difference in quinolone MICs for an *E. coli* strain with and without a *qnrA*-bearing plasmid. The report by Wang *et al*., [25],[26] showed that the MIC of ciprofloxacin increased from 0.008 µg/mL to 0.25 µg/mL in an *E.coli* J53 transconjugant with a range from 0.125 µg/mL to 2.0 µg/mL for other *qnr* plasmid transconjugants of this strain.

One study assessed the quinolone resistance conferred by 17 clinical *qnrA*-bearing plasmids. Donor bacteria originally harboring these plasmids had exhibited higher levels of resistance to quinolones than the transconjugants, suggesting that additional mechanisms of quinolone resistance frequently coexist with *qnrA*. There were also differences among transconjugants in the *qnrA* effect on fluoroquinolone MICs [15].

Although for most agents in previous studies the presence of a *qnr* plasmid increased their MIC by between 16-fold and 125-fold, this increase was less (16-fold to 32-fold) for ciprofloxacin. The agent for, which the loss of activity was least pronounced was nalidixic acid (twofold to eightfold increases in MIC) [26]. Illustrating this phenomenon, Hopkins *et al*. found that in non-Typhi *Salmonella* isolates, a phenotype of reduced susceptibility to ciprofloxacin (MIC > 0.06 µg/mL) but preserved susceptibility to nalidixic acid (MIC ≤ 16 µg/mL) identified *qnr*-positive strains [27],[28]. Also note-worthy is the finding that some *qnrA*-carrying plasmids from U.S. *K. pneumoniae* isolates yielded transconjugants with very similar quinolone susceptibilities [26], whereas other *qnrA*-carrying plasmids from U.S. and Chinese isolates of *Enterobacteriaceae* varied in ciprofloxacin susceptibilities by 16-fold [25]. There are several reasons for this phenomenon. In some cases these differences resulted from the presence of an additional resistance determinant, *aac*(*6*′)-*Ib-cr*, on some plasmids [15],[29]. For other strains, the copy number and especially the transcriptional level of the *qnr* genes affected quinolone resistance [23],[29].

*qnrS*- and *qnrB*-carrying plasmids confer quinolone resistance that is similar to that conferred by *qnrA1*. When cloned into a derivative of *E. coli* DH10B, *qnrS1* increased the MICs of nalidixic acid, ciprofloxacin, and ofloxacin 8-, 83-, and 24-fold, respectively. These changes led only to nalidixic acid resistance by CLSI breakpoints [20]. The impact of some *qnr* allele variants on quinolone MICs has additionally been examined. Overall, the patterns of resistance have been similar, with 2- to 8-fold and 8- to 32-fold increases in MICs of nalidixic acid and ciprofloxacin, respectively [20],[30],[31]

All genes isolated, amplified, sequenced and aligned in this study, were isolated from animal sources, except the two obtained from human sources. These animal sources were largely from poultry which underscores the fact that emergence of quinolone resistance genes is fast spreading via poultry. This is likely due to indiscriminate use of quinolone antibiotics as prophylaxis in poultry feed.

## 5. Conclusions

In this study, the quinolone resistance determinants- qnrA, qnrB and qnrS, were more implicated in conferring the plasmid-mediated Q-resistance and they were found on transferable plasmids which may be acquired either by conjugation or transformation. Though resistance transfer was more successful by transformation, conjugational transfer was equally possible. The *qnrB* and *qnrS* determinants were in most cases, jointly transferred and when they do, convey a higher resistance as compared with a single determinant transfer.

## 6. Recommendation

Since PMQR determinants are more observed in bacteria from animal husbandry, and these determinants are transferrable with unhealthy consequences, the following recommendations are suggested to check the FQ-resistance menace:

- The quinolones should not be used in animal husbandry for prophylactic purposes.
- Microbes from animals should not be allowed to cross-contaminate materials that will be consumed by man.
- If possible, the quinolones generally should temporarily be withdrawn from circulation to restore the potency of the drug.
